# Atomistic Structure and Dynamics of the Ca^2+^-ATPase Bound to Phosphorylated Phospholamban

**DOI:** 10.3390/ijms21197261

**Published:** 2020-10-01

**Authors:** Rodrigo Aguayo-Ortiz, L. Michel Espinoza-Fonseca

**Affiliations:** 1 Center for Arrhythmia Research, Department of Internal Medicine, Division of Cardiovascular Medicine, University of Michigan, Ann Arbor, MI 48109, USA; rodaguayo@comunidad.unam.mx; 2 Departamento de Fisicoquímica, Universidad Nacional Autónoma de México, Mexico City 04510, Mexico

**Keywords:** calcium ATPase, calcium, sarcoplasmic reticulum, membrane transport, molecular dynamics, cardiac muscle, inhibition mechanism, phospholamban, phosphorylation, structural disorder

## Abstract

Sarcoplasmic reticulum Ca^2+^-ATPase (SERCA) and phospholamban (PLB) are essential components of the cardiac Ca^2+^ transport machinery. PLB phosphorylation at residue Ser16 (pSer16) enhances SERCA activity in the heart via an unknown structural mechanism. Here, we report a fully atomistic model of SERCA bound to phosphorylated PLB and study its structural dynamics on the microsecond time scale using all-atom molecular dynamics simulations in an explicit lipid bilayer and water environment. The unstructured N-terminal phosphorylation domain of PLB samples different orientations and covers a broad area of the cytosolic domain of SERCA but forms a stable complex mediated by pSer16 interactions with a binding site formed by SERCA residues Arg324/Lys328. PLB phosphorylation does not affect the interaction between the transmembrane regions of the two proteins; however, pSer16 stabilizes a disordered structure of the N-terminal phosphorylation domain that releases key inhibitory contacts between SERCA and PLB. We found that PLB phosphorylation is sufficient to guide the structural transitions of the cytosolic headpiece that are required to produce a competent structure of SERCA. We conclude that PLB phosphorylation serves as an allosteric molecular switch that releases inhibitory contacts and strings together the catalytic elements required for SERCA activation. This atomistic model represents a vivid atomic-resolution visualization of SERCA bound to phosphorylated PLB and provides previously inaccessible insights into the structural mechanism by which PLB phosphorylation releases SERCA inhibition in the heart.

## 1. Introduction

The sarcoplasmic reticulum (SR) Ca^2+^-ATPase (SERCA) is a critical molecular component of cardiac muscle cells, regulating the rate of cardiac muscle relaxation and restoring the SR Ca^2+^ load necessary for muscle contraction in subsequent beats [1]. SERCA utilizes the energy originated from hydrolysis of one molecule of ATP to transport two Ca^2+^ ions into the lumen of the SR [2]. SERCA activity is regulated in the heart by phospholamban (PLB), a 52-residue membrane protein that resides in the SR [3]. PLB inhibits SERCA activity by decreasing Ca^2+^ affinity, and inhibition is relieved by phosphorylation, thereby regulating SERCA activity and muscle contractility [4].

PLB phosphorylation at residue Ser16 (pSer16) induces an order-to-disorder structural transition of the cytosolic domain of PLB, destabilizing a inhibitory ordered *T* state in favor of the disordered, non-inhibitory *R* state [5,6]. Two mechanisms have been proposed for the relief of SERCA inhibition by PLB phosphorylation: dissociation model and subunit model. The dissociation model proposes that upon the formation of the *R* state, PLB physically separates from SERCA to relieve inhibition [7,8] whereas the subunit model hypothesizes that PLB acts as a functional subunit of SERCA, and that the *T*-to-*R* state structural shift of PLB induced by phosphorylation relieves inhibition while preserving the SERCA–PLB heterodimer [9,10]. The two models have been supported independently by several groups, but there is solid experimental evidence that supports the subunit model because they have shown a direct interaction of PLB and SERCA upon PLB phosphorylation [11,12,13,14]. Furthermore, it is known that the cytosolic domain of PLB becomes structurally disordered upon phosphorylation, yet there has been speculation that SERCA has a well-defined pocket that binds this unstructured domain, and that this interaction is directly responsible for relief of SERCA inhibition [15]. More recent studies have shown that phosphorylation of PLB does not induce substantial changes in the interaction of the inhibitory transmembrane (TM) domain of PLB [16], thus suggesting that SERCA regulation by PLB phosphorylation is likely due to an allosteric effect exerted by the disordered cytosolic domain of PLB.

Despite these advances in the field, there are major gaps in our understanding of SERCA regulation by phosphorylation of PLB: (i) What is the atomistic structure of the regulatory complex between phosphorylated PLB and SERCA? (ii) Does SERCA have a well-defined binding site for the disordered cytosolic domain of phosphorylated PLB? (iii) What is the mechanism for control of SERCA structural dynamics by PLB phosphorylation? Answering these questions in high spatiotemporal resolution is much needed in the field to understand SERCA regulation in terms of intermolecular interactions and activities. The structure of SERCA bound to the TM domain of PLB was solved recently [17], but the cytosolic domain in the phosphorylated *R* state is intrinsically disordered, so the high-resolution structure of the of phosphorylated PLB bound to SERCA has remained inaccessible to experiments. Based on structures of the inhibitory SERCA–PLB complex and the solution structure of PLB, here we report a fully atomistic model of SERCA bound to PLB phosphorylated at residue Ser16 (SERCA–pPLB) and study the structural dynamics using microsecond-long all-atom molecular dynamics (MD) simulations in an explicit lipid bilayer and water environment. Here, we studied the complex under Ca^2+^-free conditions to study the direct effects of PLB phosphorylation alone on the functional dynamics of SERCA. The result is a realistic atomic-resolution model of the molecular interactions and mechanism by which PLB phosphorylation releases SERCA inhibition in the heart.

## 2. Results

### 2.1. Structure of the SERCA–pPLB Complex

**Figure 1:**
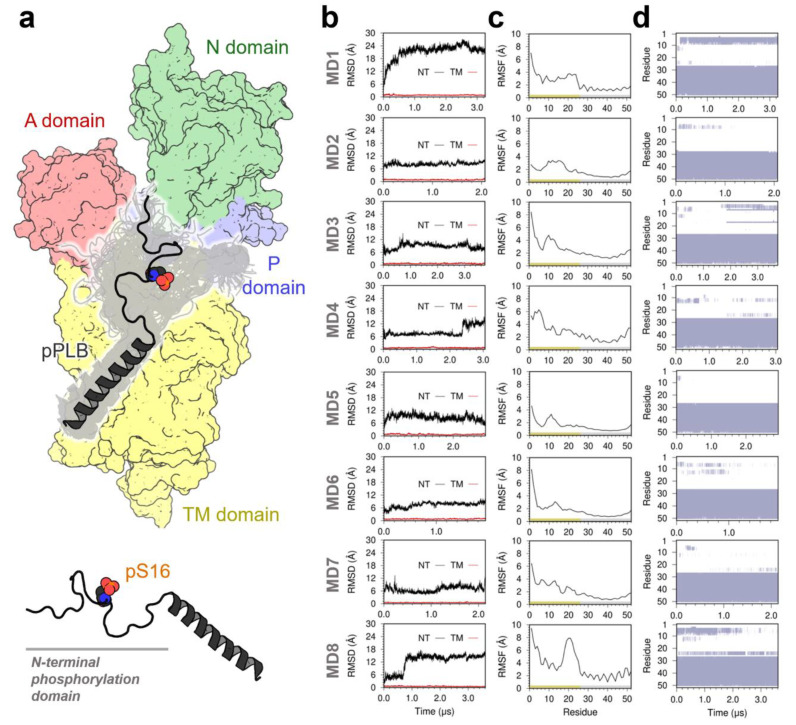
(**a**) Three-dimensional structure of phosphorylated phospholamban (PLB) bound to sarcoplasmic reticulum (SR) Ca^2+^-ATPase (SERCA). Gray ribbons represent the structures of phosphorylated PLB sampled on the SERCA–pPLB complex. SERCA is shown as a surface representation and colored according to its functional domains: A-domain, red; P-domain, blue; N-domain, green; transmembrane (TM) domain, yellow. (**b**) RMSD of the TM domain (red trace) and the N-terminal phosphorylation domain (NT, black trace) of PLB calculated from each individual molecular dynamics (MD) trajectory. RMSD was obtained by aligning the backbone of the TM domain with the structure at the beginning of each MD replicate. (**c**) RMSFs of main chain atoms for each residue of phosphorylated PLB. RMSFs were calculated from each independent MD trajectory using the backbone of the TM domain as a reference. (**d**) Secondary structure evolution of phosphorylated PLB indicating the presence of α-helical structure (violet) and unstructured regions of the protein (white).

We performed eight independent MD simulations for a total simulation time of 22.7 µs to determine the structure and stability of the SERCA–pPLB complex in the microsecond timescale. The structural ensemble of phosphorylated PLB in the MD trajectories shown in Figure 1a provides greater detail about the conformations populated in the bound complex. The structural ensemble of phosphorylated PLB in the complex is characterized by a largely unstructured N-terminal phosphorylation domain (residues 1–26) and a structurally ordered TM helix (residues 27–52). Analysis of the time-dependent RMSD of the phosphorylated PLB showed the N-terminal phosphorylation domain is flexible in the complex, with changes in the RMSD of up to 25 å compared to the initial structure of the complex (Figure 1b). Root-mean-square fluctuation (RMSF) of the main chain C_α_ atoms of the phosphorylation domain showed that this region exhibits high flexibility in the bound complex (Figure 1c). Time-dependent changes in the secondary structure showed that the N-terminal phosphorylation is largely unstructured, although the formation of transient local α-helix structure is observed in most of the trajectories (Figure 1d). Despite the disordered nature of this domain, we found that in all eight trajectories this domain settles a plateau, indicating that the structure of the cytosolic domain converges in the microsecond timescale. Conversely, RMSF values of the main chain atoms indicate that the TM helix of phosphorylated PLB has very low mobility in the complex (Figure 1c). Complementary RMSD plots showed that the TM helix rapidly settles a plateau around 1 Å in all eight trajectories, indicating that the TM domain converged to a position that is essentially identical to that of the initial structure used in this study (Figure 1b). An opposing behavior was found for the phosphorylation domain, where the TM domain has low mobility in the complex (i.e., RMSF < 2 Å, Figure 1c) and also populates a stable α-helix structure in the microsecond-long trajectories. These findings indicate that phosphorylation of PLB does not produce a change in the orientation of the TM domain in all trajectories of the SERCA–pPLB complex.

**Figure 2:**
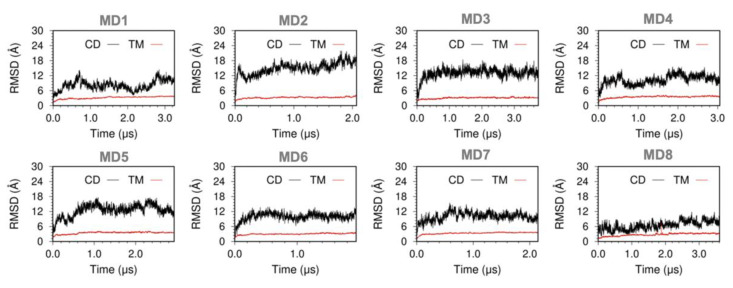
Time-dependent RMSD evolution of SERCA domains in the SERCA–pPLB complex. RMSD of the TM domain of SERCA (red trace) was calculated using backbone alignment for TM helices of the pump; RMSD of the cytosolic domain (black trace) was calculated by aligning the backbone of the cytosolic headpiece with the structure at the beginning of each trajectory.

We also calculated the backbone RMSD for the TM and cytosolic domains of SERCA to determine the structural dynamics of the pump in the trajectories. In all trajectories, the structure of the 10-helix TM domain of SERCA undergoes a rapid (t < 200 ns) drift in the RMSD (Figure 2); this small change (RMSD < 2.5 Å) in the structure can be attributed to the relaxation of the TM domain in the lipid bilayer. Following this rapid relaxation of the structure, the RMSD values remained fundamentally unchanged for the remainder of the simulation time. These findings indicate that the structure of the TM domain observed in the MD trajectories is similar to that of the initial structure built based on structures of the inhibitory SERCA–PLB complex. This suggests phosphorylated PLB does not induce sizeable changes in the overall conformation of the TM domain of SERCA. RMSD plots of the cytosolic headpiece of SERCA indicate that this domain undergoes substantial structural changes in the trajectories of the SERCA–pPLB complex (Figure 2). Previous studies have shown that the cytosolic headpiece of SERCA is inherently flexible under physiological conditions [18,19,20,21,22]; but, the RMSD plots indicate that PLB phosphorylation may induce changes in the structural dynamics of the headpiece. These effects will be discussed in Section 2.4.

### 2.2. Mapping of Interactions between the Disordered Cytosolic Domain of Phosphorylated PLB and SERCA

**Figure 3:**
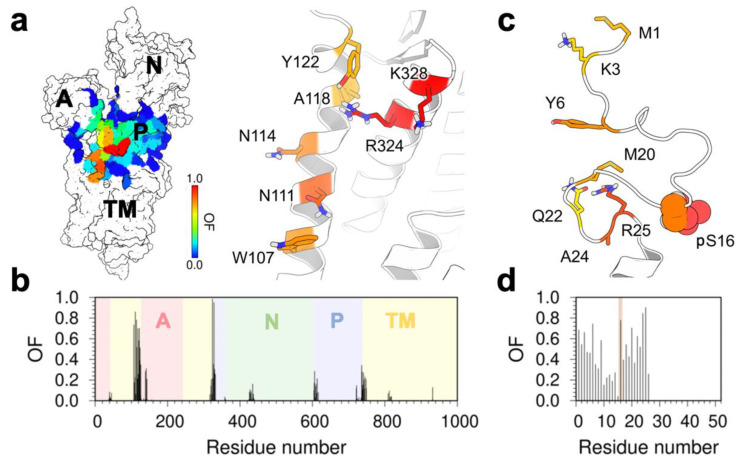
(**a**) Depiction of residues involved in the interaction of the NT domain of phosphorylated PLB with SERCA. The color scale shows the residues located within 3.0 Å of phosphorylated PLB N-terminal domain ranging from high (red) to low (blue) occupancy fraction (OF) values. (**b**) Per amino acid occupancy contacts of pPLB N-terminal domain with each SERCA domain in the simulated systems. (**c**) Phosphorylated PLB NT residues involved in the interaction with SERCA. The color scale shows the residues located within 3.0 Å of SERCA ranging from high (red) to low (blue) OF values. (**d**) Per amino acid occupancy contacts of SERCA with the NT domain of phosphorylated PLB in the simulated systems. Residue pS16 is highlighted in orange.

The structure of the SERCA–pPLB complex shows that the disordered phosphorylation domain of PLB interacts with the flexible cytosolic headpiece of SERCA. However, the simulations revealed that the N-terminal phosphorylation domain of PLB binds unspecifically to SERCA, thus contradicting previous studies suggesting that PLB phosphorylation is required to produce a relatively well-defined structure of the complex that is non-inhibitory [15]. Although a visual inspection of the complex could be sufficient to determine whether the phosphorylation domain of PLB binds non-specifically to SERCA, this approach may provide incomplete, if not inaccurate, information. Consequently, we calculated occupancy maps averaged over all trajectories to search for intermolecular interactions between the phosphorylation domain of PLB and SERCA. The phosphorylation domain of PLB interacts with all four functional domains of SERCA: namely, nucleotide-binding (N), actuator (A), phosphorylation (P), and TM domains (Figure 3a). We found that most of the interactions are formed transiently as reflected by occupancy factor values less than 0.3; transient interactions are observed primarily between the phosphorylation domain of PLB and the N-, P-, and A-domains (Figure 3b). The residues of SERCA that interact with the N-terminal phosphorylation domain of PLB include Trp107, Asn111, Asn114, Tyr122, Arg324, and Lys328 (Figure 3a), whereas PLB residues Met1, Lys3, Tyr6, pSer16, Met20, Gln22, and Arg25 interact with SERCA in the microsecond time scale (Figure 3c,d).

**Figure 4:**
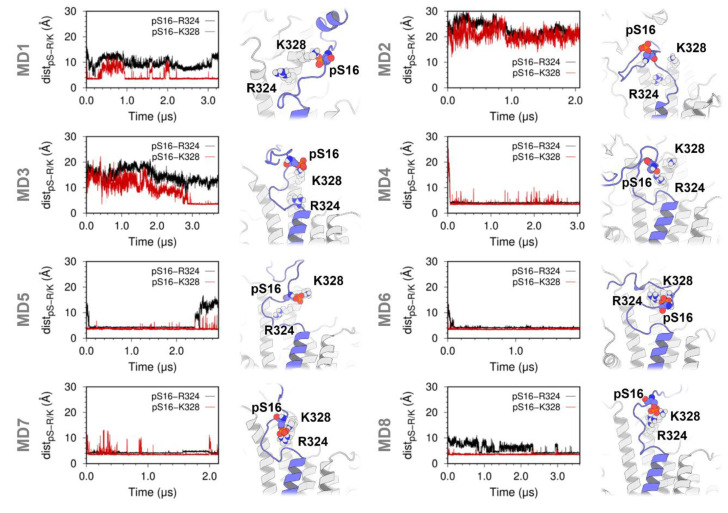
Distance evolution is shown between pSer16–Arg324 (black trace) and pSer16–Lys328 (red trace). Distances between were calculated between the phosphorous atom of pSer16 and the C_ζ_ and N_ζ_ atoms of Arg324 and Lys328, respectively. Here, we consider pSer16–Arg324 and pSer16–Lys328 pairs to form favorable interactions at distance < 6 Å [27]; the panels on the right show the structure of pSer16, Arg324, and Lys328 at the end of each MD trajectory of the SERCA–pPLB complex. SERCA and phosphorylated PLB are shown as white and purple ribbons, respectively; pSer16, Arg324, and Lys328 are shown as spheres.

The analysis showed that SERCA residues Arg324 and Lys328, as well as PLB residue pSer16 have substantially higher occupancy values (OF > 0.75) compared to other residues located at the SERCA–pPLB interface (Figure 3). Based on the complementary electrostatic properties of these residues, their accessibility to the cytosol, and their relative position in the initial structure (R = 25 Å), it is likely that these residues engage in highly specific interactions in the SERCA–pPLB complex. To test this hypothesis, we calculated inter-residue distances between pSer16 and Arg324/Lys328 of SERCA. We found that except for trajectory MD2, residue pSer16 of PLB is recruited by a cationic site of SERCA formed by residues Arg324 and Lys328. Residue Arg324 forms salt bridge interactions with the phosphate group of Ser19 in five of the MD simulations of the complex (Figure 4, trajectories MD4-MD8, red line). These residues interact either through monodentate- or bidentate-like geometries in our simulations. Nonetheless, the bidentate geometry is preferred over the monodentate one in the trajectories (Figure 4), in agreement with studies showing that the former is energetically more favorable in solution [23]. We argue that a mixture of monodentate and bidentate geometries can facilitate the reversible interaction between Arg324 and the phosphorylation site of PLB. For example, reversibility of this interaction is observed in the trajectory MD5, where the interaction between there residues is disrupted at t = 2.4 µs (Figure 4). The new salt bridge between Lys328 and the phosphate group of pSer16 is formed more frequently in the complex, and it was observed in trajectories MD1 and MD3–MD8 (Figure 4). We also found that this interaction is relatively more stable in the microsecond time scale than that formed between Arg324 and pSer16, lasting for up to 3.6 µs (e.g., trajectory MD8, Figure 4). Notably, we found that the interaction between pSer16 and Arg324/Lys328 stabilizes a disordered structure of PLB residues Thr17-Asn27 (Figure 4). This finding is important because formation of α-helical structure of residues around residue Asn27 (Lys27 in human PLB) is required for SERCA inhibition [24,25,26]; thus, stabilization of an unfolded structure by pSer16-Arg324/Lys328 illustrates the functional importance of this interaction in the SERCA–pPLB complex.

### 2.3. Effects of PLB Phosphorylation on TM Domain Interactions

**Figure 5:**
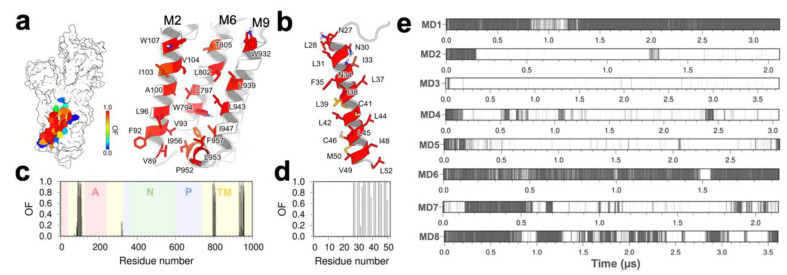
(**a**,**b**) Depiction of residues involved in the interaction of the TM domain of pPLB with SERCA. The color scale shows the residues located within 3.0 Å of pPLB NT ranging from high (red) to low (blue) occupancy factor values; (**c**,**d**) per amino acid occupancy contacts of the TM domain of phosphorylated PLB with SERCA; (**e**) time-dependent evolution of the hydrogen bond between PLB residue Asn34 and SERCA residue Gly801 calculated from each independent MD trajectory.

The crystal structure of the inhibitory SERCA–PLB complex, the TM domain of PLB binds to a canonical site formed between TM helices M2, M6, and M9 [17], and previous studies have shown that relief of PLB-induced SERCA inhibition by Ca^2+^ may occur without changes in the binding mode of PLB in the canonical site [28]. To determine if phosphorylated PLB induces changes in the interaction between the TM domain of the two proteins, we plotted occupancy maps for all residues at the SERCA–pPLB interface over the combined 22.7 µs of simulation time. We found that the canonical pocket of SERCA is predominantly occupied (OF > 0.8) by phosphorylated PLB in the microsecond timescale (Figure 5a). SERCA residues with the highest occupancy include Val89, Leu96, Ile103, and Val104 of TM helix M2, Leu802, and Thr805 of TM helix M6, and Trp932 and Leu939 of TM helix M9 (Figure 5b). Similarly, PLB residues that interact directly with the TM domain of SERCA, including Leu31, Asn34, Ile38, Leu42, Ile48, and Val49, are predicted to occupy the canonical site (OF > 0.8) upon PLB phosphorylation (Figure 5b,c). These residues establish important intermolecular interactions that are necessary for the stability of the complex between unphosphorylated PLB and SERCA [17]. Therefore, the high occupancy of these sites in the SERCA–pPLB complex suggests that PLB phosphorylation does not induce changes in the interaction between the TM domains of both proteins.

Site-directed mutagenesis, X-ray crystallography, and molecular simulations have shown that residue Asn34 of PLB plays a central role in SERCA inhibition [17,29,30]. Our occupancy analysis showed that Asn34 occupies the SERCA–PLB interface throughout the entire simulation time (OF = 1, Figure 5c,d), and this might suggest that the structural model of SERCA–pPLB reported here does not represent the non-inhibited state of the complex. We have recently shown that PLB residue Asn34 forms a stable hydrogen bond with the backbone oxygen of SERCA residue Gly801, and that formation and disruption of this interaction is a hallmark of PLB inhibition and reactivation of SERCA–PLB [28,30,31]. Therefore, we analyzed time-dependent hydrogen bonding between Asn34–Gly801 for each independent MD trajectory of the SERCA–pPLB complex. The hydrogen bond between Asn34–Gly801 is stable for more than 90% of the simulation time in only two trajectories, MD1 and MD6 (Figure 5e), but it is largely disrupted in most of the trajectories. This hydrogen bond is formed for about 58% of the time in MD7, but it is only transiently present (i.e., less than 15% of the simulation time) in trajectories MD2 through MD5 (Figure 5e).

### 2.4. Phosphorylated PLB Induces Functional Transitions in SERCA

**Figure 6:**
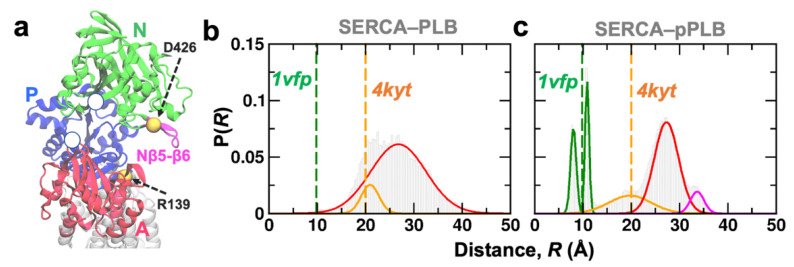
(**a**) Structure of the SERCA cytosolic headpiece showing each functional N-, A-, and P-domain in green, red, and blue, respectively. We also show the location of the functional loop Nβ5-β6 (magenta). Yellow spheres show the position of residues used to calculate interdomain distance between the A-domain and loop Nβ5-β6. Cα-Cα distance distributions between Arg139–Asp426 obtained for the (**b**) SERCA–PLB and (**c**) SERCA–pPLB complexes. Discrete distances were calculated from crystal structures of the inhibitory SERCA–PLB complex (*4kyt*, orange dashed line) and SERCA bound to Ca^2+^/AMP-PCP (*1vfp*, green dashed line).

The changes in the hydrogen bonding pattern of Asn34–Gly801 are consistent with release of SERCA inhibition and suggest that the structural ensemble of SERCA–pPLB represents the non-inhibitory state of the complex. Therefore, we asked whether disruption of the inhibitory contacts by PLB phosphorylation induces functional structural changes in the cytosolic headpiece that are associated with SERCA activation. To address this question, we first measured Cα-Cα distances between SERCA residues Arg139 (A-domain) and Asp426 (loop Nβ5-β6) (Figure 6a). We chose these residues because previous studies have shown that their interaction correlates with SERCA activity in vitro [32,33]. We excluded the first 0.2 µs from each MD trajectory to avoid biases associated with the initial configuration of the SERCA–pPLB complex. We also generated an MD ensemble of the unphosphorylated PLB bound to SERCA by combining five MD trajectories generated previously by our group into a single trajectory (total simulation time of 17.8 µs [34]). These trajectories are used as a control here to resolve precise structural changes induced by PLB phosphorylation. In the absence of PLB phosphorylation, the interresidue distance Arg139–Asp426 fits a two-Gaussian distribution, with a structural population with means at 21 Å, and a broader distribution representing a structurally dynamic population with means at 27 Å (Figure 6b). The absence of direct interaction between these residues is consistent with a catalytically incompetent structure of SERCA [32,33]. In the presence of bound phosphorylated PLB, we resolved five subpopulations of Arg139–Asp426: three structural states with means at 20, 27, and 33 Å, which are consistent the A-domain and the loop Nβ5-β6 being spatially separated, and two populations with means at 7.5 and 11 Å, which represent the binding of the A-domain and the loop Nβ5-β6 (Figure 6c). These results from our unbiased MD simulations indicate that upon PLB phosphorylation and in the absence of Ca^2+^, the headpiece in SERCA–pPLB populates structures that are distinct from those populated by the unphosphorylated SERCA–PLB complex. More importantly, this structural shift leads to a reduction in the distance between A-domain and the loop Nβ5-β6, in agreement with the notion that PLB phosphorylation stabilizes a more compact structure of SERCA’s headpiece [22].

**Figure 7:**
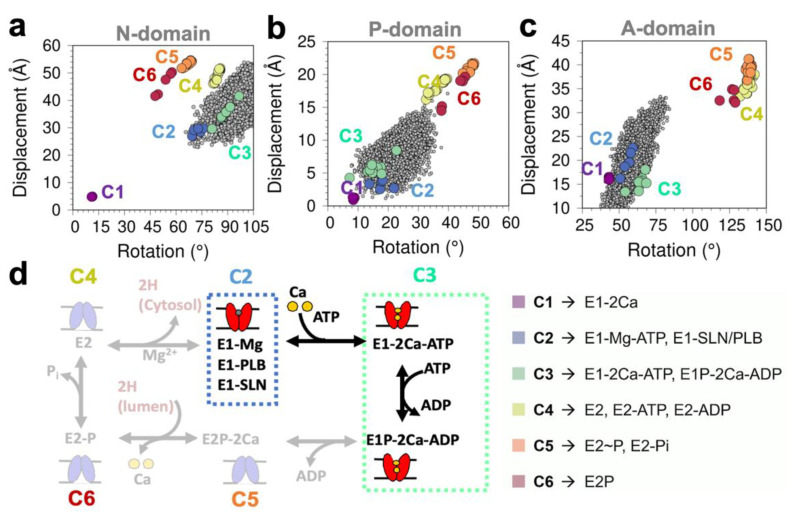
Rotation/displacement map of the (**a**) N-domain, (**b**) P-domain, and (**c**) A-domain of SERCA obtained from all trajectories combined of the SERCA–pPLB complex; the map is built based on the crystal structures of SERCA, as described in reference [36]; (**d**) schematic representation of the Post-Albers pumping cycle of SERCA linking biochemical and structural intermediates of the pump. C1–C6 represent specific biochemical intermediates obtained through clustering analysis of all available crystal structures of the pump.

We charted changes in the rotation and displacement of the N-, P-, and A-domains to gain further insight into the functional transitions of SERCA that occur in response to PLB phosphorylation. We found that both N- and P-domains, the catalytic elements of SERCA, occupy the structural space defined by the Ca^2+^/nucleotide-bound state of the pump (Figure 7a,b). This structural space includes the crystal structure of the pre-hydrolysis (cluster C2, blue circles) and post-hydrolysis (cluster C3, green circles) E1 states of the pump. Conversely, the structural map revealed that the A-domain adopts a structural arrangement similar to that of SERCA in the absence of Ca^2+^ (Figure 7c), which is defined primarily by crystal structures of SERCA bound to Mg^2+^, PLB, and the regulatory protein sarcolipin (cluster C2, blue circles). The absence of a Ca^2+^-bound-like orientation of the A-domain in the trajectories of SERCA–pPLB is consistent with binding of Ca^2+^ ions and correct charge neutralization of the transport sites is required for the formation of a competent orientation of this domain [35]. Overall, the structural shifts in the catalytic N- and P- domains in the SERCA–pPLB complex are generally similar to those required for activation of SERCA, thus indicating that PLB phosphorylation alone is sufficient to populate a non-inhibited state of the pump (Figure 7d).

## 3. Discussion

We present an atomistic structure of phosphorylated PLB bound to SERCA and its structural dynamics under Ca^2+^-free conditions, thus providing new insights into the mechanism for regulation of SERCA by PLB phosphorylation. In agreement with EPR and NMR spectroscopy studies [9,15], MD simulations showed that the N-terminal domain of phosphorylated PLB is largely unstructured in the complex, but it forms a stable heterodimeric complex with SERCA in the microsecond timescale. The interaction of the disordered N-terminal phosphorylation domain of PLB with SERCA is enabled primarily by the phosphate group of pSer16. This interaction also facilitates burial of PLB residues Lys3 and Tyr6, in agreement with NMR spectroscopy studies showing that ^13^C resonances of these residues are almost completely quenched upon PLB phosphorylation by spin-labeled SERCA at position Cys674 [15]. Furthermore, we found that PLB phosphorylation does not disrupt the heterodimeric SERCA–pPLB complex in the microsecond timescale, and also does not induce measurable changes in the orientation of PLB’s TM helix in the complex. This observation is important because we used the crystal structure of the inhibited SERCA–PLB complex, and our simulations validate previous EPR spectroscopy studies suggesting that the TM domain phosphorylated PLB binds to SERCA in an orientation similar to that of the inhibitory unphosphorylated PLB [16]. The agreement between experiments and simulations provide solid evidence that the unbiased simulations reported in this study recapitulate the intrinsic structural features of the SERCA–pPLB complex in the cell.

NMR spectroscopy studies have suggested that upon phosphorylation, the N-terminal phosphorylation domain of PLB interacts with a preferred binding pocket located between the P- and N-domains of SERCA [15]. Contrary to conclusions in these studies, we found that the disordered N-terminal domain does not bind to a well-defined region of the SERCA headpiece, and instead samples different orientations and covers a broad area of the cytosolic domain of SERCA that includes all three N-, P-, and A- domains. Nonetheless, our analysis revealed the formation of stable electrostatic interactions involving pSer16 of PLB and residues Arg324/Lys328 of SERCA. We found that the interaction between Arg324/Lys328 and pSer16 is characterized by diverse salt-bridge configurations. This structural heterogeneity of this interaction is likely a functional feature of this interaction and likely contributes to both the adaptability of the complex as well as to the reversibility of the regulatory SERCA–pPLB interaction at physiological conditions [37]. We also found that this pSer16-mediated interaction stabilizes an unfolded structure of residues Thr17-Asn27, thus increasing the structural disorder of this region and favoring the disruption of key intermolecular contacts between SERCA and PLB [24,25,26]. Structural order in this region has been proposed to participate in important SERCA–PLB inhibitory contacts [38], thus further supporting the functional role of Arg324/Lys328 in PLB phosphorylation-mediated release of SERCA inhibition. The functional interaction between pSer16 and Arg324/Lys328 also helps explain previous findings showing that phosphorylation facilitates binding of PLB’s N-terminal phosphorylation domain to SERCA [15,39]. Both Arg324 and Lys328 are highly conserved in mammalian species and have been associated with important regulatory functions that include signal transduction and coupling of SERCA [40,41]. Owing to the functional importance of these residues and their intrinsic electrostatic properties at physiological conditions, we propose that Arg324/Lys328 likely constitutes the canonical binding motif required to stabilize the heterodimeric SERCA–pPLB complex.

Atomistic simulations showed that the SERCA–pPLB complex is stable in the microsecond time scale, so we asked whether PLB phosphorylation disrupts key inhibitory interactions between the two proteins. We focused on an intermolecular hydrogen bond between the side chain of PLB residue Asn34 and the backbone oxygen of SERCA residue Gly801 because this interaction is required for SERCA inhibition. In two of the MD trajectories of SERCA–pPLB, the complex forms a hydrogen bond between PLB residue Asn34 and SERCA residue Gly801. These hydrogen bond, which is an essential intermolecular contact responsible for PLB inhibition of SERCA [25,28,30,31,38], remains intact for most of the simulation time in these trajectories. Nevertheless, we found that this inhibitory interaction is disrupted in most of the MD trajectories of the SERCA–pPLB complex. In these cases, the initially formed hydrogen bond formed between Asn43 and Gly801 is present transiently in the trajectories, and several hydrogen bond formation and disruption events are observed in the time scales used here. Our dynamic structural model also showed that PLB phosphorylation acts upon Asn34–Gly801 and renders the hydrogen bond interaction between these two residues unstable. Although these structural changes have not been directly observed in experimental studies, our findings are consistent with previous studies showing disruption of this interaction is a hallmark of Ca^2+^-mediated relief of SERCA inhibition by PLB [28]. It is also worth noting that previous studies showed that Ca^2+^ binding to SERCA–PLB completely breaks the inhibitory interaction Asn34–Gly801 in the microsecond timescale, but the effect of PLB phosphorylation is not as robust as that induced by Ca^2+^ binding [28]. This observation suggests that while PLB phosphorylation alone disrupts the inhibitory contact between Asn34 and Gly801, Ca^2+^ binding in the transport sites is still required to induce complete disruption of this interaction. Nevertheless, destabilization of this interaction in the absence of Ca^2+^ agrees with the notion that PLB phosphorylation acts as a molecular switch that releases SERCA inhibition and further demonstrates that the structural model obtained in this study corresponds to that of the non-inhibited state of the regulatory complex.

Finally, we ask whether PLB phosphorylation induces specific structural changes that are associated with the formation of a competent structure of SERCA. We found that in the SERCA–pPLB complex and upon release of the inhibitory contacts between Asn34/Gly801, SERCA residues Arg139 of the A-domain, and Asp426 of loop Nβ5-β6 come close together. This structural shift is similar to that found in the crystal structure of the activated Ca^2+^/ATP-bound complex [42], and it is important for early formation of the catalytically active structure of SERCA [32]. Moreover, we found that even in the absence of bound Ca^2+^, PLB phosphorylation is sufficient to guide the structural transitions of the N- and P-domains that are required to produce a competent structure of SERCA. These findings are in agreement with live-cell FRET experiments showing that PLB phosphorylation induces a redistribution of SERCA structural states to produce a more compact and catalytic competent structure of the pump [22]. It is reasonable to argue that pSer16 serves as a molecular anchor or tether that brings together the A-domain and loop Nβ5-β6 and stabilizes this bound conformation via favorable electrostatic interactions. However, we found that the phosphorylation site of PLB interacts primarily with a Arg324/Lys328, and that this site is located ~30 Å away from the A-domain–loop Nβ5-β6 interface. Based on this evidence, we propose a mechanistic model in which PLB phosphorylation relieves interdomain inhibitory contacts between SERCA and PLB and also serves as a mediator of allosteric signaling that favorably orients and strings together the catalytic elements required for ATP utilization, thus dictating the structural transitions required for regulation of Ca^2+^ transport in the heart [20,32,33,42].

In summary, we obtained an atomistic model of the SERCA–pPLB complex, mapped the interactions between the two proteins, and determined the effects of PLB phosphorylation on the functional dynamics of SERCA. We found that the unstructured N-terminal phosphorylation domain samples different orientations and covers a broad area of the cytosolic domain of SERCA, but it forms a stable complex via pSer16 interactions with a binding site formed by SERCA residues Arg324/Lys328. The dynamic nature of the interaction helps explain the adaptability and reversibility of SERCA–PLB inhibition, thus constituting a functional feature intrinsic of the regulatory mechanism. PLB phosphorylation does not affect the interaction between TM domains of SERCA and PLB. However, PLB phosphorylation releases inhibitory contacts within the complex and guides the structural transitions that are required to produce a competent structure of SERCA. We conclude that PLB phosphorylation serves as an allosteric molecular switch that releases inhibitory contacts and strings together the catalytic elements required for SERCA activation. Based on these findings, we provide novel hypotheses that can be tested by functional mutagenesis studies to validate the putative SERCA site Arg324/Lys328, and also by complementary spectroscopy and simulation studies [34] to establish the structure-function relationships of SERCA regulation by PLB. The mechanistic insights from this study will likely have important implications for our understanding of the molecular switches that underly regulation of Ca^2+^ transport, and will also help understand the structural features required for therapeutic modulation of SERCA in the heart.

## 4. Materials and Methods

### 4.1. Construction of the Initial Structure of SERCA Bound to Phosphorylated PLB

We used the structure of the full-length structure of the *R* state of monomeric AFA-PLB (Cys36Ala, Cys41Phe, Cys46Ala) obtained using solution NMR (PDB: *2lpf*, [43]) as the initial model to build the SERCA–pPLB complex. First, we aligned residues 28-51 of the solution NMR structure onto residues 27–50 of PLB4 in the crystal structure of SERCA–PLB (PDB: *4kyt*, [17]). Upon alignment, we combined the NMR structure of the *R* state of PLB with the crystal structure of SERCA–PLB to produce an initial model of the complex. Finally, residues Ala36, Phe41, and Ala46 of AFA-PLB were changed to alanine to produce a model of native PLB. We performed initial 100-ns MD simulations of the complex in a 1-palmitoyl-2-oleoyl-*sn*-glycero-3-phosphocholine (POPC) bilayer. The stereochemical quality of the initial models was verified with PROCHECK [44,45], showing no major aberrations in the geometry of the complexes. In this initial model, the cytosolic domain of PLB (Met1-Gln26) is intrinsically unstructured, whereas the TM domain (Asn27-Leu52) populate an α-helical structure. Furthermore, we found that cytosolic residues Met1-Leu7 and Gln22-Gln26 of PLB directly interact with SERCA, whereas residues Thr8-Pro21 (which includes pSer16) are primarily exposed to the solvent. This model was used as the starting conformation to simulate the dynamics of SERCA–pPLB.

### 4.2. Preparation of the SERCA–pPLB Complex

We used the atomistic model of the full-length PLB bound to SERCA generated here study to simulate the SERCA‒pPLB complex under Ca^2+^-free conditions. We modeled residue pSer16 of PLB, and SERCA residues Glu309 and Asp800 as unprotonated and residues Glu771 and Glu908 as protonated. In addition, we adjusted the p*K*_a_ of other ionizable residues to a pH value of approximately 7.2 using PROPKA [46,47]. The complex was inserted in a pre-equilibrated 130 × 130 Å bilayer of POPC lipids. We used the first layer phospholipids that surround SERCA in the E1 state [48] as a reference to insert the complex in the lipid bilayer. This initial system was solvated using TIP3P water molecules with a minimum margin of 30 and 25 Å in the z-axis between the edges of the periodic box and the cytosolic and luminal domains of SERCA, respectively. K^+^ and Cl^-^ ions were added to neutralize the system and to produce a KCl concentration of ~100 mM.

### 4.3. Molecular Dynamics Simulations

MD simulations of all systems were performed with NAMD [49] using periodic boundary conditions [50], particle mesh Ewald [51,52], a non-bonded cutoff of 12 Å, and a 2-fs time step. CHARMM36 force field topologies and parameters were used for the proteins [53], lipid [54], water and ions. The NPT ensemble was maintained with a Langevin thermostat (310 K) and an anisotropic Langevin piston barostat (1 atm). Fully solvated systems were first subjected to energy minimization, followed by gradually warming up of the systems for 200 ps. This procedure was followed by 10 ns of equilibration with backbone atoms harmonically restrained using a force constant of 10 kcal mol^−1^ Å^−2^. We performed 8 independent MD simulations of SERCA–pPLB for a total simulation time of 22.7 µs.

### 4.4. Structural Analysis and Visualization

We calculated the backbone root mean square deviation (RMSD) for SERCA, cytosolic, and TM domains, and PLB, N-terminal phosphorylation, and TM domains. Changes in the secondary structure of PLB was computed employing the program *dssp* [55] included in the GROMACS package [56]. We also calculated the occupancy fraction (OF) of SERCA residues located within 3.0 Å of PLB and PLB residues located within 3.0 Å of SERCA using the *select* built-in tool implemented in GROMACS [56]. The presence of a hydrogen bond between the amide group of Asn34 and the backbone oxygen of Gly801 was calculated using *HydrogenBondAnalysis* library of MDAnalysis. We mapped MD structures onto the rotation-displacement map created using 74 crystal structures of SERCA to determine the effects of PLB phosphorylation on the structural dynamics of the cytosolic domain [36]. All SERCA structures were aligned to the Cα atoms of crystal structures *2c9m* [57] using the following TM domain helices as reference: M7 (residues 831–855), M8 (residues 895–915), M9 (residues 933–948), and M10 (residues 966–994). Translation distances and rotation angles of the N-, P-, and A-domains were computed for the three cytosolic domains using *2c9m* [57] as reference with the *draw_rotation_axis.py* script running on PyMOL (http://pymolwiki.org/index.php/RotationAxis). The programs PyMOL and VMD [58] were used for visualization and rendering of the structures.

## Abbreviations

A-domain	Actuator domain
EPR	Electronic Paramagnetic Resonance
FRET	Fluorescence Resonance Energy Transfer
MD	Molecular dynamics
N-domain	Nucleotide-binding domain
NMR	Nuclear Magnetic Resonance
P-domain	Phosphorylation domain
PDB	Protein Data Bank
PLB	Phospholamban
pPLB	Phosphorylated phospholamban
POPC	1-palmitoyl-2-oleoyl-*sn*-glycero-3-phosphocholine
RMSD	Root-mean-square deviation
RMSF	Root-mean-square fluctuation
SERCA	Sarcoendoplasmic reticulum Ca^2+^-ATPase
SR	Sarcoplasmic reticulum
TM	Transmembrane
